# Modulation of PARP-1 Activity in a Broad Time Window Attenuates Memorizing Fear

**DOI:** 10.3390/ijms22126170

**Published:** 2021-06-08

**Authors:** Einat Elharrar, Yahav Dikshtein, Sapir Meninger-Mordechay, Yehuda Lichtenstein, Gal Yadid

**Affiliations:** 1 Leslie and Susan Gonda (Goldschmied) Multidisciplinary Brain Research Center, Bar-Ilan University, Ramat-Gan 52900, Israel; einatosh@gmail.com (E.E.); sapirmn16@gmail.com (S.M.-M.); yehudalicht@gmail.com (Y.L.); 2 The Mina & Everard Goodman Faculty of Life Sciences, Bar-Ilan University, Ramat-Gan 52900, Israel; yahavosg@gmail.com

**Keywords:** PARP-1, PTSD, central amygdala, anxiety, memory consolidation, fear conditioning

## Abstract

The amygdala plays a critical role in the acquisition and consolidation of fear-related memories. Recent studies have demonstrated that ADP-ribosylation of histones, accelerated by PARPs, affects the chromatin structure and the binding of chromatin remodeling complexes with transcription factors. Inhibition of PARP-1 activity during the labile phase of re-consolidation may erase memory. Accordingly, we investigated the possibility of interfering with fear conditioning by PARP-1 inhibition. Herein, we demonstrate that injection of PARP-1 inhibitors, specifically into the CeA or i.p., in different time windows post-retrieval, attenuates freezing behavior. Moreover, the association of memory with pharmacokinetic timing of PARP inhibitor arrival to the brain enabled/achieved attenuation of a specific cue-associated memory of fear but did not hinder other memories (even traumatic events) associated with other cues. Our results suggest using PARP-1 inhibitors as a new avenue for future treatment of PTSD by disrupting specific traumatic memories in a broad time window, even long after the traumatic event. The safety of using these PARP inhibitors, that is, not interfering with other natural memories, is an added value.

## 1. Introduction

Pavlovian aversive conditioning is one of the most commonly used paradigms to investigate the neurobiological basis of emotion, learning and memory [1]. During the acquisition phase, a neutral stimulus is paired with an aversive unconditioned stimulus to consolidate a new memory. This memory exists for a limited time in a labile state until stabilization-progress, namely, memory consolidation, and becomes a long-lasting memory [2]. Memories are inscribed as stable traces in the brain; however, once they are retrieved, they are rendered labile and can be modified during the progress of reconsolidation [3]. Disrupting or facilitating the reconsolidation of emotional memories by stress exposure has important implications for understanding anxiety disorders linked to traumatic memories, such as post-traumatic stress disorder (PTSD) [4]. Several studies indicate that by reconsolidation blocking, fear memories can be weakened [5].

The amygdala is part of the limbic system and is considered essential in the formation and storage of emotional memories [6,7]. It is hypothesized that in fear conditioning, the basolateral amygdala (BLA) supports the formation of conditioned fear memory and projects to neurons in the central nucleus (CeA) for the expression of conditioned fear responses [8,9]. The CeA, the main amygdaloid output structure, sends projections to various autonomic and somatomotor centers involved in mediating specific fear responses [10]. Lesions of CeA neurons abolish the expression of conditioned freezing and fear-potentiated startle [11].

Poly(ADP-ribose) polymerases (PARPs) are a family of cell signaling enzymes in the cell nuclei [12]. PARP-1 modifies various nuclear proteins by poly(ADP-ribosyl)ation, which has a role in gene transcription through several mechanisms, including de-condensation of chromatin, activation of particular transcription factors, and modulation of transcription regulatory complexes, in addition to its involvement in DNA repair [13,14]. Goldberg et al. (2009) [15] indicated that polyADP-ribosylated PARP-1 and phosphorylated extracellular signal-regulated kinase (ERK) interacted with each other by a positive feedback mechanism, enhancing and prolonging PARP-1 polyADP-ribosylation and up-regulating ERK activity in the nucleus, including phosphorylation of ERK target transcription factor Elk1 [16,17]. Elk1 phosphorylation up-regulated the CREB binding protein histone acetyltransferases activity (and thereby histone acetylation) and the expression of Elk1 target genes, including immediate early genes that are implicated in LTM [18,19]. In previous research, we demonstrated the effect of PARP-1 activity in the CeA on cocaine-associated memory and conditioned place preference (CPP) with cocaine. We also demonstrated that inhibition of PARP-1 activity during cocaine-associated memory retrieval abolished CPP; compared to inhibition after memory retrieval, which did not affect the CPP reconsolidation process and subsequent retrievals [20].

In a review done by Brewin about new methods to block traumatic memories, it was discussed that biological studies on memory consolidation and reconsolidation attempt to affect the initial establishment of traumatic memory and avoid the development of PTSD. This attempt remains inconclusive in terms of practicality and effectiveness. On the other hand, when focusing on the reconsolidation hypothesis, the use of propranolol seem more appropriate among patients with PTSD show encouraging results [21].

Prolonged exposure (PE) is a therapy method for PTSD, aims to dissociate the memories of a traumatic event from their negative valence [22]. PE requires the patient to re-live their traumatic experiences repeatedly within a safe context in a process referred to as “flooding.” Constant exposure to the traumatic thoughts decoupled from actual threat can induce extinction of the trauma response [23]. In a review on PE, it was proposed that initiation of forgetting could reduce the length of the PE therapy and accelerates it. This is because constant and repetitive traumatic retrieval is not essential for the improvement of neglecting traumatic memories, when using modulators that enhance the generation of neurons in a specific tissue compartment or ‘neurogenic niche’ occupied by their parent stem cells (neurogenesis). Hence, interventions that encourage forgetting may represent a different method for altering traumatic memories [24]. Our study aims to examine a method that combines a single re-living the traumatic experiences with a modulator of the machinery of cellular memory.

In the current study, we used a fear-conditioning paradigm pairing between a tone as a natural emotional stimulus and a foot shock to investigate the PARP-1 activity of fear memory in the central amygdala. We used two potent PARP-1 inhibitors, PJ-34 and ABT-888, to test the role of PARP-1 modulation in fear conditioning. The primary aim was to test as a proof of concept whether PARP inhibitors could be used as a future treatment for PTSD. Using a model of fear condition, we gave i.p. PARP inhibitor in a broad time window in the context of memory reactivation (Experiment 1). Once the concept was established, we had three goals: 1. We aimed to define specific brain regions for such PARP ability suggested for PTSD pathology. For this purpose, we injected PARP inhibitors, associated with the traumatic cues (reminders), to the CeA and BLA within a short time window, nearby the initial traumatic event, as suggested by physicians [25]. High dose hydrocortisone immediately after trauma may alter the trajectory of PTSD: interplay between clinical and animal studies) (Experiment 2); 2. We tested the effect of PARP inhibitors when applied long after the traumatic event (Experiment 3); 3. We examined our ability to direct PARP inhibition to erase the selected harmful traumatic event, leaving other memories unaffected. We first examined whether the erased descript memory is associated solely with the trauma event by testing the intactness of the natural memory learning machinery (Experiments 4A). Then, we examined the ability to learn further emotional (even traumatic) memories after erasing a traumatic event by testing the integrity of the physiological system after intervention with PARP inhibitors. We applied exposure to cat litter scent for the traumatic event (Experiment 4B).

## 2. Results

### 2.1. Effect of Inhibition of PARP-1 Activation on Fear Conditioning

**Figure 1:**
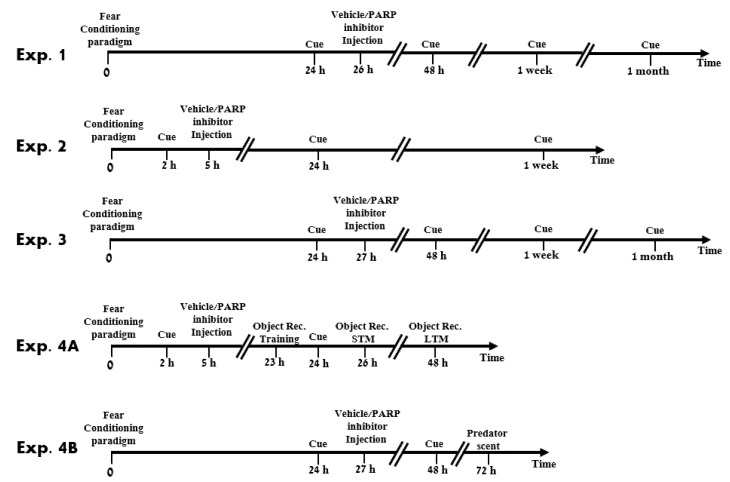
Flow chart of experiment procedures. Experiment 1: Rats were trained to the fear conditioning procedure. A day later, they were cued with a tone and, after 2 h, they were injected i.p. with aCSF/PARP inhibitor (ABT-888) (15 mg/kg) one time, and behavioral performance was tested. To test long term erasure, the rats were audibly cued again 24 h, 1 week, and 1 month after the trauma, and their behavioral performance was tested again. Then, the rats were euthanized, and their brains were excised. Experiment 2: Rats were trained to fear conditioning procedure. Two hours later, they were audibly cued, and after 3 h, they were injected with aCSF/PARP inhibitor (PJ-34/ABT-888) (50 µM/10 µM, respectively) and behavioral performance was tested. The rates were audibly cued again 1 day and 1 week after the trauma, and their behavioral performance was tested. Then, the rats were euthanized, and their brains were excised. Experiment 3: Rats were trained to the fear conditioning procedure. Twenty-four hours later, they were audibly cued, and after 3 h, they were injected with aCSF/PARP inhibitor (PJ-34/ABT-888) (50 µM/10 µM, respectively) into the CeA or the BLA. The rats were audibly cued again 1 day, 1 week, and 1 month after the trauma, and their behavioral performance was tested. Then, the rats were euthanized, and their brains were excised. Experiment 4A: Rats were trained to the fear conditioning procedure. Two hours later, they were audibly cued, and after 3 h, they were injected with aCSF/PARP inhibitor (PJ-34) (50 µM) into the CeA or the BLA. The next day, the rats were trained for the object recognition test, and an hour later, they were audibly cued. After 2 h, the rats were tested for their object recognition STM and after 1 day, for their LTM. Experiment 4B: Rats were trained to fear conditioning procedure. Twenty-four hours later, they were auditory cued, and after 3 h, they were injected with aCSF/PARP inhibitor (ABT-888) (10 µM, respectively). The rats were audibly cued again after 1 day, and their behavior was recorded. Twenty-four hours later, the rats were exposed to another trauma—predator scent—for 30 min. After exposure to the trauma, each rat was transferred to a clean open field arena, and its behavioral parameters were measured.

**Figure 2:**
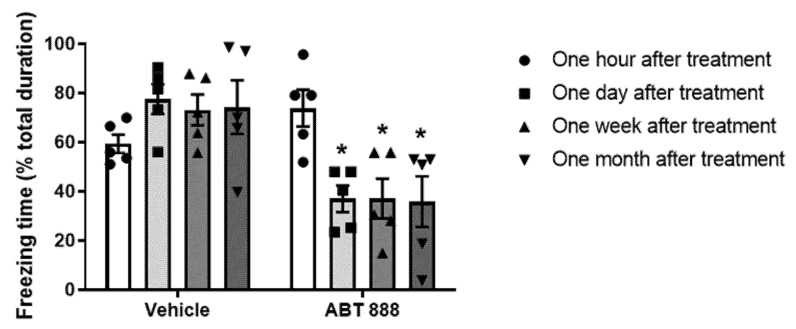
(Exp. 1). A cue was presented 24 h after the training, followed by a single I.P. injection of PARP inhibitor disrupts fearful memory. Rats were given i.p. injection of PARP inhibitor ABT-888 (*n* = 10; 15 mg/kg) 2 h after one auditory fear conditioning trial and 1 day after the fear conditioning learning. The rats were tested 24 h and 1 week after the cue-tone presentation. Inhibition of PARP-1 activity showed attenuation of fear long-term memory (LTM): 1 day, 7 days, and 1 month post-cue (two-way ANOVA; * *p* < 0.05). The corresponding group showed no difference in the freezing behavior (two-way ANOVA; * *p* < 0.05). The figure depicts the mean freezing levels + SEM during the LTM.

ABT-888 is a potent inhibitor of PARP that can cross the blood–brain barrier and is highly effective at suppressing PARP activity, evident by the reduced level of PARP modifications (15 mg/kg i.p.) [26]. A group of rats was trained to fear according to the fear conditioning paradigm. Twenty-four hours later, a tone, that is, a cue, was presented in a different context, that is, “Open field”, and the freezing behavior of the animal was measured for 5 min. After 2 h, the PARP-inhibitor ABT-888 or the vehicle aCSF (control) was infused intraperitoneal (i.p.) (15 mg/kg). The next day, the effect of the treatment was assessed by another cue presentation, and freezing behavior was again measured. The rats were tested again seven days and one month after the Vehicle or PARP inhibitor infusion (see Exp. 1, in Figure 1). Analysis of the freezing behavior showed that ABT-888 i.p injection reduced the freezing behavior compared to control (Figure 2), indicating that PARP-inhibitors alter the response to a memory of a traumatic event (cue-tone). 

### 2.2. Effect of Inhibition of PARP-1 Activation on Fear Conditioning Immediately after Training

A group of rats was surgically implanted with a cannula in the CeA (as described in the Methods Section 4.3, location of injection is shown in Figure 8). After rehabilitation, rats were trained to fear using the fear conditioning paradigm. Two hours later, a tone (cue) was presented, and freezing behavior of the rats was measured. After three hours, PARP-inhibitors (PJ-34 or ABT-888) or aCSF were infused into the amygdala: CeA (central) or BLA (basolateral amygdala); two groups for each region. The next day, the treatment effect was assessed by another cue presentation, and PTSD-like behavior of the rats was measured. The rats were tested again seven days after the Vehicle/PARP inhibitors infusion (see Exp. 2, Figure 1).

To define specific brain regions for the PARP-inhibitor effect, we implanted four groups of rats with a guide cannula: two into the CeA, and other two into the BLA (as described in the Methods Section 4.3). Then, we subjected the rats to the fear conditioning training procedure.

After 14 days, the fear conditioning training was performed, and we tested their post-conditioning memory retrieval in the “open field” apparatus. Three hours later, the rats (in both CeA-implanted and BLA-implanted groups) were infused via the cannula with either the PARP inhibitors, PJ-34 or ABT-888, or an equivalent volume of the vehicle (aCSF).

**Figure 3:**
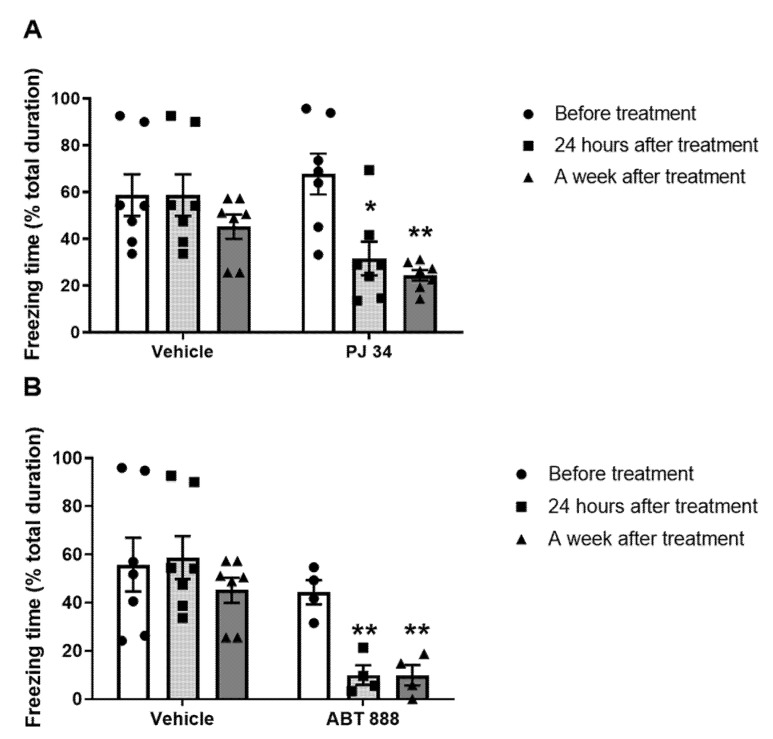
(Exp. 2). A single injection of PARP1 inhibitors into the Central Amygdala (CeA) shortly after the training disrupts fearful memory. (**A**): Rats were given micro-infusions of PARP inhibitor, PJ34 (50 μM; 1.2 μL; *n* = 7) into the central amygdala (CeA) 3 h after one auditory fear conditioning trial. The rats were tested 24 h and 1 week after the cue-tone presentation. Inhibition of PARP activity showed attenuation of fear in long-term memory (LTM) 1 day and 7 days post-cue (two-way ANOVA; * *p* < 0.01, ** *p* < 0.01). The corresponding group showed no difference in the freezing behavior (*p* > 0.5). (**B**): Rats were given micro-infusions of PARP inhibitor, ABT-888 (10 μM; 1.2 μL; *n* = 7) into the central amygdala (CeA) 3 h after one auditory fear conditioning trial. The rats were tested 1 day and 7 days later after the cue-tone presentation. Inhibition of PARP activity showed attenuation of fear in long-term memory (LTM) 1 day and 7 days post-cue (two-way ANOVA; * *p* < 0.05, ** *p* < 0.01). The corresponding group showed no difference in the freezing behavior (*p* > 0.5). The figure depicts the mean freezing levels ± SEM.

**Figure 4:**
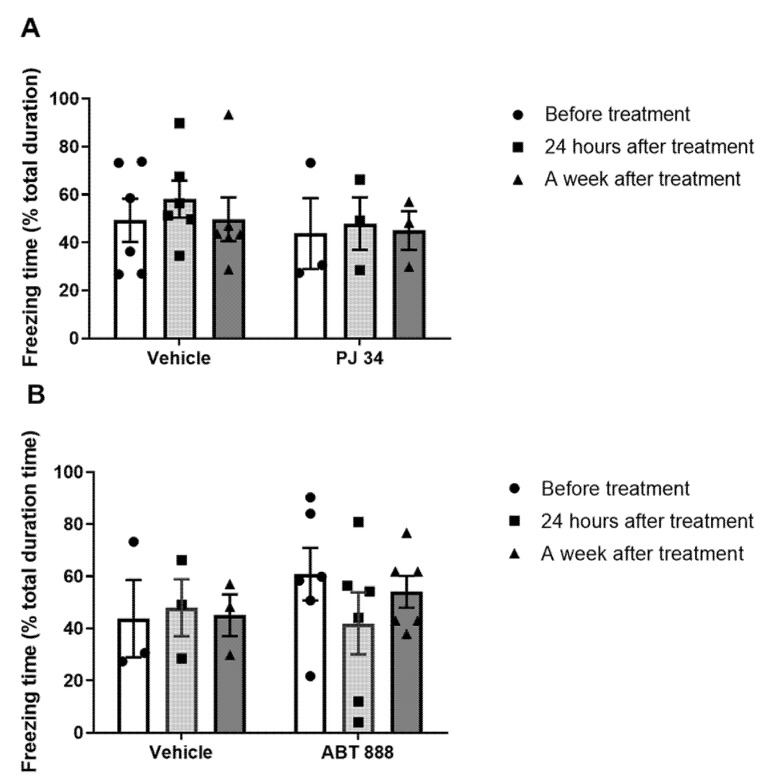
(Exp. 2). Single injection of PARP inhibitors into the Basolateral Amygdala (BLA) shortly after the training does not affect fearful memory. (**A**) Rats were given micro-infusions of PARP inhibitors, PJ34 (50 μM; 1.2 μL *n* = 7) into the basolateral amygdala (BLA) 3 h after one auditory fear conditioning trial. The rats were tested 1 day and 7 days after the cue-tone presentation. Inhibition of PARP activity showed no effect on long-term memory (LTM) for fear conditioning when tested 1 day and 7 days later. (Two-way ANOVA; *p* < 0.05). (**B**) Rats were given micro-infusions of PARP inhibitor, ABT-888 (10 μM; 1.2 μL; *n* = 6) into the basolateral amygdala (BLA) 3 h after one auditory fear conditioning trial. The rats were tested 24 h and 1 week after the cue-tone presentation. Inhibition of PARP activity showed no effect on long-term memory (LTM) for fear conditioning when tested 1 day and 7 days later (two-way ANOVA; *p* < 0.05). The figure depicts the mean freezing levels ± SEM.

Two-way ANOVA revealed that freezing behavior was abolished in rats infused with PJ-34 or ABT-888 into the CeA; this remission lasted for the next seven days (Figure 3A,B, respectively). In contrast, in rats injected with PJ-34 or ABT-888 into the neighboring region, BLA did not show a group effect (Figure 4A,B, respectively).

### 2.3. Long Term Effect of Inhibition of PARP-1 Activity on Fear Conditioning—A Day after Training

A different group of rats was surgically implanted with a cannula to CeA. After rehabilitation, rats were trained to fear using the fear conditioning paradigm. Twenty-four hours later, tone (cue) was presented and freezing behavior of the rats was measured. After three hours, PARP-inhibitor (ABT-888) or aCSF was infused into the central amygdala (CeA) as described in the previous Section 2.2. The effect of the treatment was assessed on the following day by presenting the auditory cue followed by monitoring the freezing behavior of the animals immediately and again at 7 days and one month (see flow chart in Figure 1).

**Figure 5:**
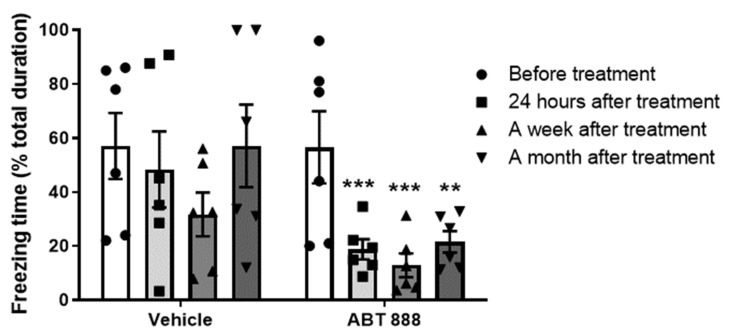
(Exp. 3). A cue was presented 24 h after the training, followed by a single injection into the Central Amygdala of PARP inhibitor disrupts fearful memory even for one month. Rats were given micro-infusions of PARP inhibitor ABT-888 (10 μM, 1.2 μL; *n* = 10) into the central amygdala (CeA) 3 h after one auditory fear conditioning trial and 1 day after the fear conditioning learning. The rats were tested for behavioral performance 24 h, 1 week, and 1 month after the cue-tone presentation. Inhibition of PARP-1 activity showed attenuation of fear in long-term memory (LTM) 1 month post-cue (two-way ANOVA; ** *p* < 0.01, *** *p* < 0.001). The figure depicts the mean freezing levels ± SEM during the LTM.

Analysis confirmed that the memory of the traumatic event could be erased a day after the trauma, and this fear erasure may last for at least another month (Figure 5).

### 2.4. Directing PARP-1 Inhibition to a Specific Memory

To rule out the possibility that infusion of the PARP inhibitor into the CeA impairs natural (not associated with the specific traumatic event) memory formation, we tested the object-recognition abilities of PJ-34-treated and untreated rats immediately after the fear conditioning procedure (see Exp. 4A, Figure 1).

#### 2.4.1. Effects on Natural Spatial Memory (Object Recognition Test)

**Figure 6:**
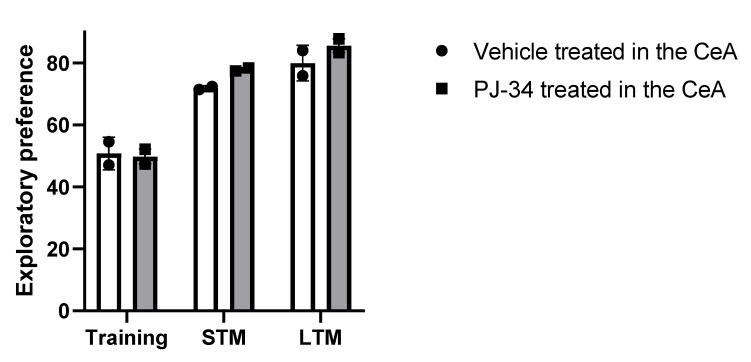
(Exp. 4A). A single injection of PARP inhibitor into the Central Amygdala (CeA) does not affect STM and LTM of object recognition test. Object recognition in an open field was assessed to rule out the possibility of damage to short-term and long-term object-recognition memory (STM and LTM, respectively) due to treatment with the inhibitor. Rats were given micro-infusions of PARP inhibitor (PJ34) (50 μM, 1.2 μL; *n* = 8) into the central amygdala (CeA) 3 h after one auditory fear conditioning trial. Then, the rats were exposed to two identical objects for 5 min. Three hours later, a short-term memory (STM) test was carried out. Long-term memory (LTM) was tested 24 h after training. No differences in exploratory preferences over either the short term (tested 1.5 h after training) or the long term (tested 24 h after training) were observed between rats treated by infusion of PJ-34 into the CeA and by infusion of vehicle into the CeA (*p* > 0.5, one-way ANOVA). Data are presented as mean ± SEM of the percentage of time spent exploring a particular object divided by the total time of object exploration.

The rats were subjected to object recognition tests for the assessment of short-term (STM) and long-term memory (STM). The mean time spent interacting with each object did not differ significantly between rats infused with PARP inhibitor (PJ-34) and rats treated with vehicle Figure 6). These findings confirm that treatment with the inhibitor does not impair either short-term or long-term object-recognition memory.

#### 2.4.2. Effect of Attenuation of PARP Activity on a Following Different Traumatic Event (Predator Scent)

To test the effect of PARP-1 inhibition on acquisition of new and different traumatic events, we exposed rats to the same paradigm as described before, and after erasing its memory, we exposed them again to a new and different of trauma—a predator scent. For this, we used another group of rats trained with the fear conditioning paradigm, as described above. Twenty-four hours later, tone (cue) was presented, and freezing behavior of the rats was measured for 5 min. After three hours, PARP-inhibitor (ABT-888) or aCSF was applied. The next day, the effect of the treatment was assessed by monitoring the rats’ behavior post-tone presentation. A day later, the rats were exposed to another trauma, that is, predator scent. Individual rats were placed for 30 min in a clean plastic cylinder containing 125 mL of well-soiled cat litter used by a cat during the 24 h prior to the experiment. The rats were exposed to the predator scent for about 30 min. After exposure to the trauma, each rat was transferred to the open field, and its behavior was measured. In the open field, rats were consecutively tested for a post-startle response after exposure to loud noise (“hyperarousal”; 5 min). The loud noise was a 36,570.3 dB pick/scale A over baseline noise of 5570.5 dB measured by Quest instrument, Quest Technologies, model 2900, calibrated by QC-10 calibrator 114 dB–1000 Hz, which was sounded to the tested rats for the first five minutes of hyperarousal. (The two types of traumas utilize different types of sound). The cylinder was cleaned between the testing of each rat (Exp. 4B, Figure 1).

**Figure 7:**
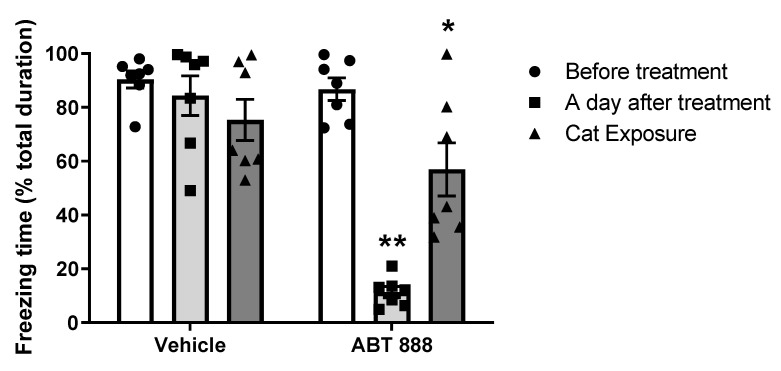
(Exp. 4B). A cue was presented 24 h after the training, followed by a single injection into the Central Amygdala does not disrupt fearful memory of a different trauma. Rats were given micro-infusions of PARP inhibitor ABT-888 (*n* = 10; 10 μM; 1.2 μL) into the central amygdala (CeA) 3 h after one auditory fear conditioning trial and 1 day after the fear conditioning learning. Inhibition of PARP-1 activity showed significant attenuation of fear post-cue (two-way ANOVA, ** *p* < 0.01). Twenty-four hours later, the rats were exposed to a litter with a cat scent for 30 min. A significant difference was shown in freezing behavior during the hyper-arousal condition for treated rats with PARP inhibitors 24 h after the treatment compared with rats after the new traumatic event (two-way ANOVA, * *p* < 0.05, ** *p* < 0.01). The analysis showed no significant differences in the behavior of the treated rats with aCSF 24 h after the treatment nor after the new traumatic event (*p* > 0.05). Bars represent Mean ± SEM. *n* = 7 per group.

When the tone was presented a day after the traumatic event, rats demonstrated a significant attenuation in freezing behavior in the “open field” compartment of PARP inhibitors. Nonetheless, twenty-four hours later after the exposure to the cat scent, we observed a significant difference in freezing behavior between the treated rats 24 h after treatment vs. their state after the exposure to the cat scent. This suggests that PARP inhibition is strictly associated with retrieval memory of a trauma event and does not prevent memorizing later different trauma events (Figure 7). 

## 3. Discussion

Posttraumatic stress disorder has significant cognitive symptoms that cannot be adequately treated by current psychopharmacological tools. Several animal models have been proposed for elucidating the neurobiological mechanisms involved in the etiology of this disease, including the behavioral paradigm of fear conditioning in which associative learning processes are involved in modifying the response to a conditioned stimulus [15,27,28,29].

Using cue based fear conditioning [25,27,28,30], we have found that pharmacological inhibition of PARP activity in situ, by injecting its inhibitor into the CeA or by applying it IP, obliterated the animal’s traumatic response to the fear-associated cue. Our intervention time windows were chosen due to the pharmacokinetics, derived from several examples in the literature. Zohar et al. [25] suggested application of a high-dose of hydrocortisone no longer than the first few hours after a traumatic experience, for beneficial results. From this study and other animal studies, scientists proposed that there is a “window of opportunity” in the early aftermath of trauma to help those who are vulnerable to the development of PTSD. In another example, propranolol (a non-selective beta blocker, used to treat hypertension, anxiety and panic) administered immediately after trauma exposure reduced PTSD severity two months later [30,31,32]. We aimed to expand this short “window of opportunity”, in order to try and help those who did not manage to receive the treatment in the few “golden hours” after the traumatic experience. This is in accordance with Ledoux and his collaborators who suggested that in order to treat patients suffering from PTSD efficiently, administration of propranolol or other drugs will have to be given in the context of traumatic memory reactivation, and not necessarily during an early time window [33]. Our findings indicate that administration of PARP inhibitor in the context of memory reactivation blocks reconsolidation of the aversive memory by precisely targeting the CeA, in a wide time range window after the traumatic event.

The amygdala is an important brain structure that plays a role in the acquirement and expression of conditioned fear responses. When it comes to the parts of the amygdala, the CeA is thought to be mainly involved in behavioral manifestation of conditioned fear responses [34]. Therefore, we sought to investigate the effect of PARP-1 inhibition on the CeA. Neither further short-term nor long-term spatial memory formation (tested by object recognition task) [35] as well as exposure to further different trauma (predator scent) were altered following infusion of PARP inhibitor into the CeA, indicating that this memory easement was of a specific association with the certain trauma and not a global easement, supporting its safe potential for clinically use.

It was shown that PARP-1 activity in the hippocampus and the medial prefrontal cortex (mPFC) is required for consolidation and reconsolidation of contextual fear memory, as well as for its long-term extinction [36]. Thus, attenuation of fear memory may be enabled by accessing these brain areas. Nonetheless, we postulate that for long term effect, interference with PARP activity is necessary in the CeA.

It has been suggested that PARP-1 activation in molecular processes underlies long-term memory formation during learning [15,29]. ADP-ribosylation of the linker histone H1 and activation of PARP-1 were associated with long-term facilitation and associative memory in Aplysia [29]. This goes along with Szyf and colleagues’ previous hypothesis that alterations in DNA epigenetics serve as a “genomic” memory of physiological and cognitive performance [37]. In a previous study we reported that PARP-1 is highly enriched in genes involved in various aspects of brain function, as well as in the acquisition and retrieval of cocaine-associated memory [20]. Due to PARP-1 epigenetic properties, along with our current results, we suggest conducting future research regarding possible epigenetic regulation in the CeA, in order to detect potential biomarkers associated with PTSD-like manifestation. Identifying the epigenetic consequences of programming may provide epigenetic biomarkers for early diagnosis of disease. These may potentially serve to recognize susceptible individuals at risk, and promote the development of novel preventive and curative clinical interventions [38]. Interestingly, recent pivotal data implicated reduction in transposase inhibitor expression, caused by PARP-1 binding, in leading to elevated transposase activity and therefore induction of DNA transposition [37].

In this specific research we did not address the possibility of PARP inhibition for treating PTSD-like symptoms in females, and this should be addressed in future experiments. Furthermore, emerging data suggest that PTSD affects not only the victims’ physical and mental health but also those of their friends and family members. It was shown that families with a member suffering from PTSD experienced low relationship pleasure, incidents of familial violence and a negative effect on the family members’ mental health [39]. This issue and other important issues of diverse PTSD such as late-onset PTSD (PTSD that primarily erupts six-months or more after the occurrence of the traumatic event) [40] and complex PTSD (PTSD that occurs as a result of exposure to chronic, multiple or repeated traumas, that an escape from them is considered difficult or even impossible) [41] should also be tested for the effect of PARP inhibition.

In summary, PTSD is characterized by an inability to extinguish emotional fear memories. Since PARP-1 inhibitors appear to enhance the extinction of fear, targeting impaired extinction in anxiety disorders, such as PTSD, may prove an important and novel approach to enhance treatment efficacy. Our results provide strong evidence for the role of PARP inhibition in the CeA as a potential intervention affecting emotional long-term memory of fearful tasks.

The demonstrated ability of PARP inhibitors to disrupt memory appears to be safe, as it is specific to certain cues- induced memory-retrieval of a discerning traumatic event. Moreover, some clinical trials with PARP inhibitors already showed the potential efficacy for treating various brain diseases such as advanced solid brain tumors [42], and ischemic brain injury [43,44]. This potentially opens a novel therapeutic capacity for other brain diseases, possibly including PTSD.

To conclude, our results present the possible use of PARP-1 inhibitors as a new avenue for future treatment for PTSD by disrupting specific traumatic memories in a broad time window, even long after the traumatic event. The safety of using these PARP inhibitors, that is, not interfering with other natural memories, is an added value.

## 4. Materials and Methods

### 4.1. Subjects

Male Sprague-Dawley rats (250–300 g) were purchased from Harlan Inc. (Indianapolis, IN, USA). The animals were maintained under conditions of constant temperature (23 °C) and humidity (50%) in a 12:12 h light/dark cycle, with free access to food and water. All animal procedures were approved by the Bar-Ilan University “Institutional Animal Care and Use Committee (IACUC)” in approved protocols number 12 February 2016 and 29 May 2020, and were carried out in accordance with the National Institutes of Health Guide for the Care and Use of Laboratory Animals. 

### 4.2. Behavioral Procedures

#### 4.2.1. Fear Conditioning Model

For cue and contextual fear conditioning, rats were placed in the fear-conditioning apparatus for 3 min (using predator scent method, previously described in an article by Gal Warhaftig et al. [7], and then a 30-s tone (2.8 kHz, 65 dB) was delivered. At the last second of the tone, a 1 s shock (1.5 mA) was applied to the floor grid. This protocol was repeated three times with 2 min between pairings. The stimulus strength and the number of training pairs were chosen based on pilot experiments to optimize learning. This training was repeated three days consecutively. To assess cue learning, the animals were placed in a different context (novel food odor, cage floor, and visual cues) 24 h after training. Baseline behavior was measured for 2 min in the novel context, and then the tone was presented for 1 min continuously. Learning was assessed by measuring freezing behavior (i.e., motionless position). The behavior of the rat during testing was recorded on a computer-based event recorder. EthoVision (Noldus Information Technology, Wageningen, The Netherlands) was used for offline analysis.

#### 4.2.2. Locomotor Test

Fear-conditioned rats were infused intracranial with PJ-34 (hydrochloride hydrate, Merck KGaA, Darmstadt, Germany), ABT-888 (NSC 737664, da-ta Biotech LTD Rehovot, Israel), or vehicle, as described above, and were then placed in an open field (60 × 60 cm) apparatus with 30-cm-high walls. Following 30 s tone, their locomotor activity in the open field was measured. 

#### 4.2.3. Object Recognition Test

The same open-field apparatus was used for the object-recognition task [45], which was performed by cocaine-conditioned rats following their intracranial infusion of PJ-34 or vehicle, as described above. Four objects (M, N, P, and R) were used: the “M” and “N” objects were two identical plastic apples, “P” was a plastic ball, and “R” was a plastic rat. All were similar in texture, color patterning (red, green, yellow) and size (8 cm long and 8 cm high) but had distinctive shapes. In a training session conducted 1 h after habituation to the arena (10 min), the rat was placed for 5 min in the field, in which two identical objects (objects M and N) had been positioned in two adjacent corners, 10 cm from the walls. Short-term memory (STM) was assessed, 1.5 h after the training session, by analysis of the rat’s exploration of the open field for 5 min in the presence of one familiar (M) and one novel object (P). “Exploration” was defined as sniffing or touching the object with the nose and/or forepaws. The exploratory preference for each object was calculated as the time spent in exploring that object, expressed as a percentage of the total exploration time [(N/(N + M) × 100%]. Short-term memory recognition was evaluated as the time spent by the rat in exploring the novel object, expressed as a percentage of the total exploration time [STM = (P/(M + P) × 100%]. Between trials, the objects were washed with 10% ethanol solution. In the test of long-term memory (LTM) conducted 24 h after the training session, the same rat was allowed to explore the field for 5 min in the presence of the same familiar object (M) and a novel object (R). LTM recognition was evaluated as (R/(M + R) × 100%].

### 4.3. Surgery and Cannula Implantation

To investigate how inhibition of PARP-1 activation affects fear conditioning, rats were anesthetized intraperitoneally (i.p.) with ketamine hydrochloride (100 mg/kg) and xylazine (10 mg/kg). A guide cannula (30 gauge) was implanted 1 mm above the CeA (in two groups of rats) or the BLA (one group), sealed with a cannula dummy (Plastics One, Roanoke, VA), and secured to the skull with screws and dental acrylic cement. Coordinates of the cannula relative to bregma [46] were as follows: CeA: anterior–2.56, lateral–4, ventral–8 mm; BLA, anterior–2.8, lateral–5, ventral–8.5 mm. Rimadyl (2 mg/kg) was injected (i.p.) after surgery. The rats were allowed to recover from surgery for 10–14 days before undergoing the fear conditioning procedure.

### 4.4. Intracranial Infusions

The potent PARP inhibitor PJ-34 (N-(6-oxo-5,6-dihydrophenenthridin-2-yl)-*N*,*N*-dimethyl-acetamide; Alexis, Lausanne, Switzerland), 50 µM or ABT-888 (Veliparib), 10 µM was added to an artificial cerebrospinal fluid vehicle (aCSF) consisting of 126 mM NaCl, 2.4 mM CaCl_2_, 1.2 mM KCl, 1.2 mM MgCl_2_, 1.2 mM NaH_2_PO_4_, and NaHCO, pH 7.4. The solution (total volume 1.2 µL) was infused for 5 min, 3 h after reactivation of a consolidated fear memory producer, into the CeA or BLA using an electronic syringe pump (CMA 400, CMA/Microdialysis). Control rats received similar infusions of 1.2 µL of the vehicle only. The internal cannula remained in place for 5 min after the infusion.

### 4.5. Intraperitoneal Injection

The potent PARP inhibitor ABT-888 (Veliparib), 15 mg/kg was added to Dimethyl sulfoxide (DMSO, 0.035%). The time of injection and the concentration were chosen by the pharmacokinetic properties of the compound [47,48]. The solution was infused i.p. about 2 h after reactivation of a consolidated fear memory producer.

### 4.6. Verifying the Location of Intrabrain Injection

On conclusion of the fear conditioning experiment, rats were anesthetized and perfused transcranial with phosphate-buffered saline (PBS) followed by 4% paraformaldehyde. Their brains were removed and immersed in 4% paraformaldehyde for 24 h, and then in a phosphate buffer with 30% sucrose for 48 h. They were then frozen on dry ice and sliced (40-μ sections) with a cryostat microtome. Sections were mounted on glass slides coated with 2% gelatin, stained with cresyl violet and placement of the cannula was verified under a microscope.

### 4.7. Histology

**Figure 8:**
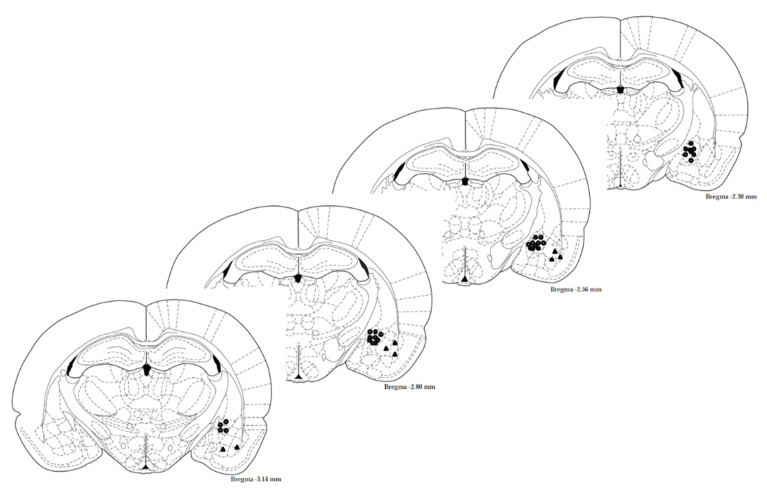
Cannula location. A diagram presenting the histological staining proving the exact location of the insertion of a cannula into the CeA or BLA region of the brain.

For verification of guide cannula placement, rats were anesthetized on conclusion of the behavioral experiments and transcardially perfused with phosphate-buffered saline (PBS) followed by 4% paraformaldehyde. Brains were removed and immersed in 4% paraformaldehyde for 24 h, and then in a phosphate buffer with 30% sucrose for 48 h. The brains were then frozen on dry ice and sliced (40-μ sections) with a cryostat microtome. Sections were mounted on glass slides coated with 2% gelatin, stained with cresyl violet, and placement of the cannula was verified under a microscope (Figure 8).

### 4.8. Statistical Analysis

Two-way ANOVA with repeated measures followed by Bonferroni’s post-hoc test was performed for analysis of all the behavioral results.

## Data Availability

Data is contained within the article.
